# Supplementary material for: Chromothripsis during telomere crisis is independent of NHEJ, and consistent with a replicative origin
Source: Genome Res. 2019 May;29(5):737–49. doi: 10.1101/gr.240705.118 (PMC6499312; doi:10.1101/gr.240705.118)
Supplement: Supplemental Material [file supp_gr.240705.118_Supplemental_file_1.zip › contigs/annotated_contigs/DB115/contig.4.DB115_length_1429_mean_cov_13.6983904829.docx]

**DB115_length_1429_mean_cov_13.6983904829**

```
ACTGGGACCTCAGTTCCACAACCATAAGAAGCTGAGGTATGCCAATAAATATTGGGCTCAGAAGATGATTTTCCCCAAAATCTTCCATG
 >chr2:22104410-22104680 + E=3e-150 p=1e-02
TAAGAACTCAGACAATCTCATACCTTGATTTCAACTTTGTGATACTTTAAGCAGAGAACTCGGTCATTCATGCTAGGCTTCTGATTTAC

AGAACTGTGAGCTAATAAATGAGTGTTACACATGTATAGTTTTTAAACATTTAAACAATTTACAAAAATAAGATTCTGGGACAATAGAG

ACC|GTAAGGGTAGCAGACAGAATGCAACCTAGAGAAGAACTGGCAGTTGGTAAGGGACCAAGGTGAAGGAGAATGTATTTTTCACCCA
     >chr2:22107360-22107823 - E=3e-266
TAAAGTTCAGTAGTGCTTTTAGAATTGCAAATATGCATTTAAATTTAATAATACAATTATGGTATATTAAATGTCAAATGACTGTTACA

GAAAAGCACTTAAAATTAATACATATGCATGAGATAAATCCTGTCTGGTTGTCAAGGTCTGAATTCAAAGTTATATAAGCCAGGCTTTG

CACTGAATTTTGTTACATCTTAAAGTTGCAAAGATCTTTGAATTTCATTGGTATTTAGCAAAGAAGCATGACCAAAGCTTAGTTAGAAC

TGAGAGAAAAGGTGAGATTTTCGGGCAGTCAGCAGCAAAACCCAAATGCTATTCAAATCCAAAGTAAAACTGAAAGCAATAGTCATCTT

CATTGCTGCTGTCATCATTGTC|C|ATATATATATATATATGGAAGGAATTATCTAGGGACTTCCTATCATCTGGCCTAATATTTTCCT
                        >chr2:22105648-22106007 - E=2e-196
GTAGTAAGGTATCCAAAAAGCAAGAAACGTAGCTCCTCATATCTTTTTTTTTTAACACTTCATGATAATCGGAGTAATTGCAGAGTGAT

ATCCATTGATCCTCATTTACAGTTTGAGAGTTTGTAATATATAAGTAATCATCAGTTACTGATAAATTCCTCTTTTCCACCATCAATCT

GTACTTGGACAAACCAGCAATCCTGGGGAAGCATAGCCACTTGTAGACCCAAAACTGTGGACTGTAGAGGGAGCAGACAATATTAGTGA

ATCTTCTTGGCTGGAGATAAAAATA|TA|TACCCTTACCCCGATGGCATTCCCTCTTCTTTCCTCAACCTCAAATTCTCAACTTAGTTT
                            >chr2:22107814-22108151 + E=2e-190
CTCTTTCAATTTTTTAATGTGTTCCCCTCTCCTCTCTCTGTGATTCATGGTCATTCACTCTCAACTTCATCTTTCTTCTTCTGGCATGG

AAATGTCCTTTGCTTCTGTTTTCTCCAATAGTCTCTCATGCAGTCAAATCCCTCACTCCCCTGTGGAGCAGTTTCAGGATGCTGCAGTG

ACTTTGACTCATCATCCATGAGCTTTAACCATAGTCCAGGGGAGCATTTTCCAAACATTAAAATGACCTGTCATTGAAAACGGCTTTCT

TAAGTGGTAA
```
